# Supplementary material for: Precise allele-specific genome editing by spatiotemporal control of CRISPR-Cas9 via pronuclear transplantation
Source: Nat Commun. 2020 Sep 14;11:4593. doi: 10.1038/s41467-020-18391-y (PMC7490392; doi:10.1038/s41467-020-18391-y)
Supplement: Supplementary file 1 — Supplementary Information [file 41467_2020_18391_MOESM1_ESM.pdf]

Supplementary Information for

Precise allele-specific genome editing by spatiotemporal control of CRISPR-Cas9 via pronuclear transplantation

(Li et al.)

Includes:

Supplementary Figure. 1-8

Supplementary Table. 1-5

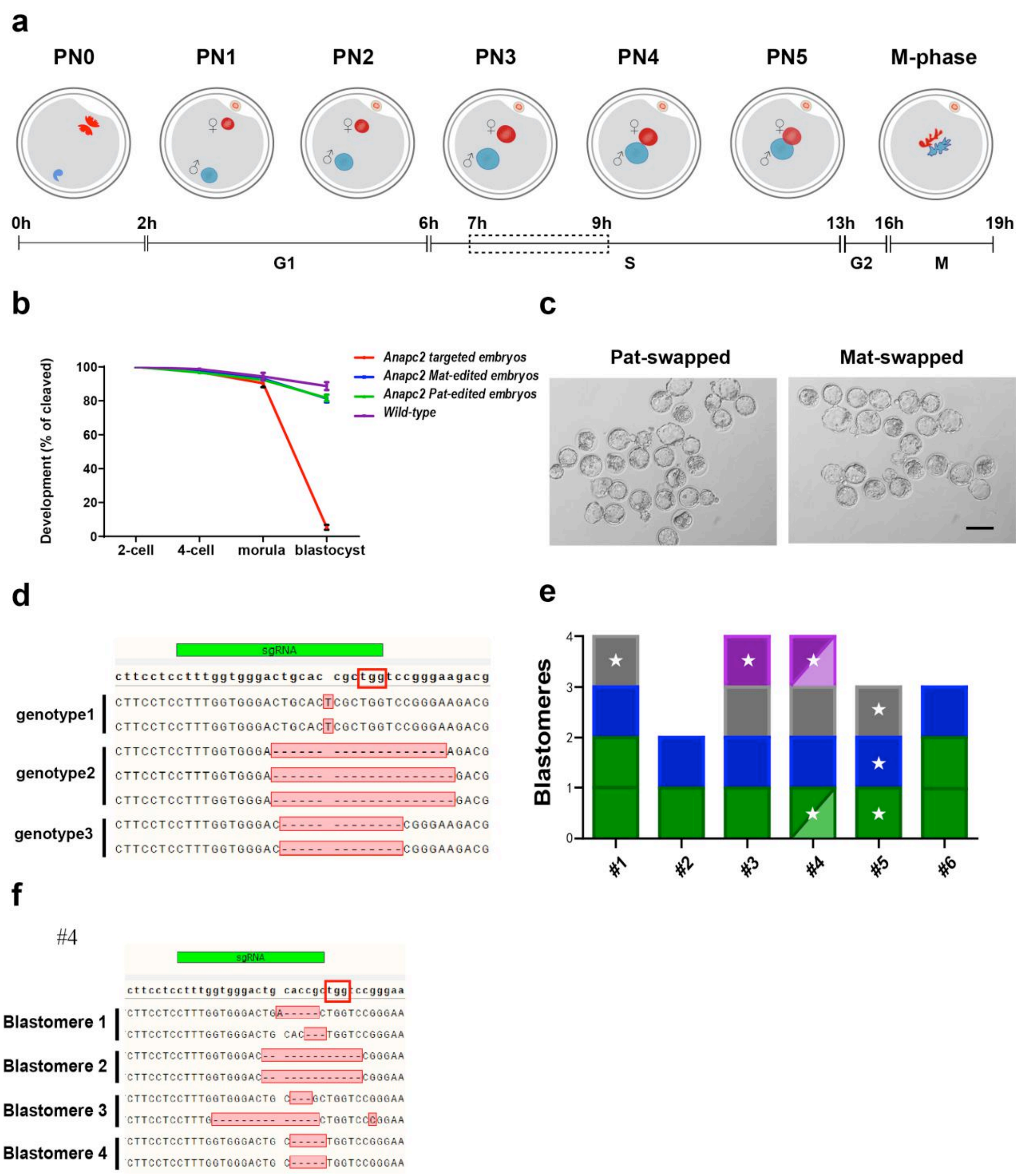

Supplementary Figure 1. **Development and genotype of Anapc2 targeted embryos. a** Schematic overview zygotic cell cycle. The dashed box represents the time for pronuclear isolation. **b** The pre-implantation developmental potential of *Anapc2* targeted embryos, *Anapc2* Pat/Mat targeted embryos and wildtype embryos followed until the blastocyst stage. *Anapc2* targeted embryos were arrested at morula stage. But *Anapc2* Pat/Mat targeted embryos can develop to blastocyst stage. Shown is the percentage of embryos that reach each indicated stage. Error bars reflect mean ± s.e.m. of n=3 independent experiments. Source data are provided as a Source Data file. **c** Bright fields of mat/pat-swapped wildtype embryos One representative result of three independent experiments is

shown. Scale bars: 100 µm. **d** TA clone sequences of the *Anapc2* sgRNA target sites from one of the zygote injection embryos. Sequence of 7 alleles present in this embryo reveals three types of mutation alleles. **e** Individual blastomere genotyping outcomes in 6 *Anapc2* one-cell injection embryos at the 4-cell stage. Each color represents a mutation genotype in each blastomere. Blended color represents a blastomere with different genotypes. White star represents genotypes without frame shift mutations (See Supp. Table 2 for details). **f** Single-cell sequencing for the No. 4 4-cell stage embryo. Biallelic sequencing outcomes of every blastomeres were shown. There exists two different indels in the biallelic targeting sites of blastomere1 and 3. Each blastomere has a different mutant genotype from each of others in the No.4 embryo (See Supp. Table 2 for details).

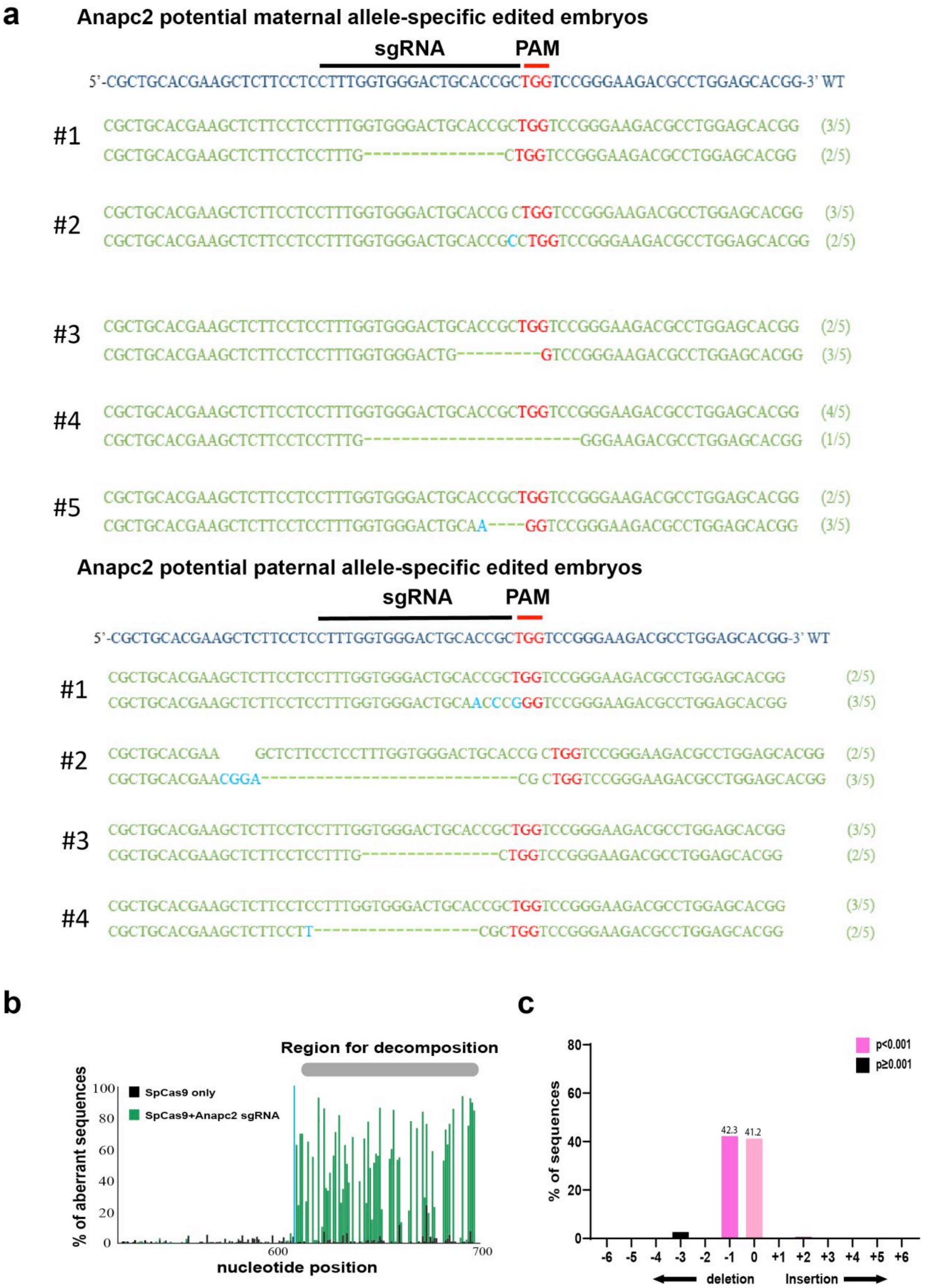

Supplementary figure 2. Genotypes of Anapc2 targeted embryos generated by Past-CRISPR. **a** TA cloning sequences of the *Anapc2* gRNA target sites from five *Anapc2* potential maternal allele specific edited embryos and four *Anapc2* potential paternal allele specific edited embryos. Sequence of several alleles present in individual embryo reveals two different types of alleles, half of them

are wild-type alleles and another half are mutant alleles. **b** Sanger-sequencing data were analyzed by TIDE. The genome edited embryo sample (SpCas9+*Anapc2* sgRNA) and the control embryo sample (SpCas9 only) are overlaid. Downstream of the expected cut site (blue full line) the percentage of aberrant sequences was quantified in the region for decomposition (See methods for details). **c** The sanger-sequencing data of the single embryo edited by Past-CRISPR were analyzed by TIDE. Decomposition of the mixed PCR production yielding the spectrum of indels and frequencies shows just one deletion indels and wildtype genotypes and the equal frequency between them.

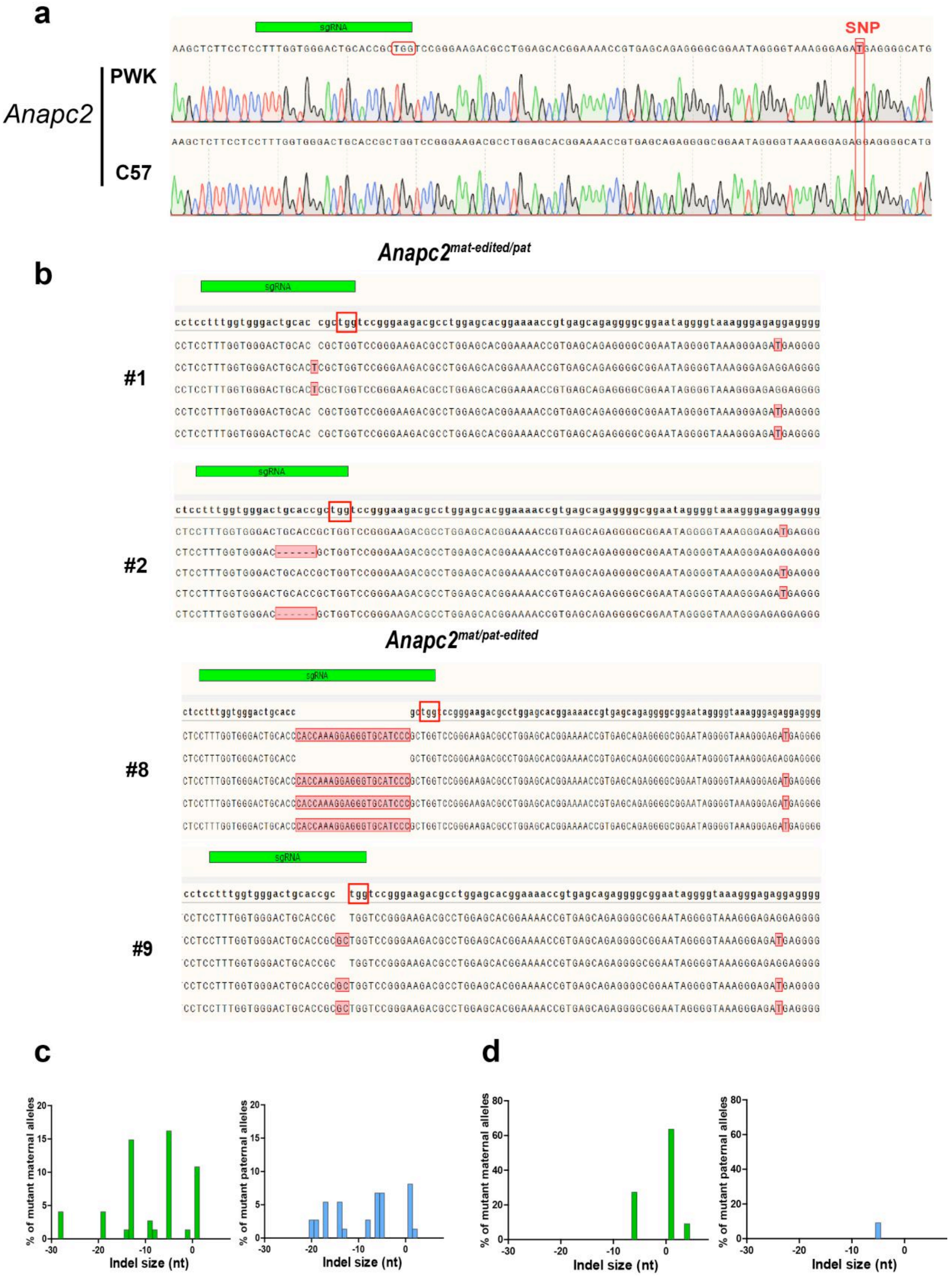

Supplementary figure 3. Matching outcomes between parental allelic mutation sites and parental respective SNPs. a The SNP site between C57BL/6J and PWK strains is indicated by red box. **b** Sequence of alleles present in several *Anapc2* mat-edited/pat and *Anapc2* mat/pat-edited hybrid embryos. All the C57 alleles contain mutational sites but none of the PWK alleles contain mutational sites in the *Anapc2* mat-edited/pat hybrid embryos, and all the PWK alleles contain

mutational sites but none of the C57 alleles contain mutational sites in the *Anapc2* mat/pat-edited hybrid embryos. c Indel profiles of embryos injected with Cas9 and sgRNA targeting Anapc2. Indel profiles of Anapc2 maternal alleles (left) and paternal alleles (right) are plotted separately. Minus numbers represent deletions and plus numbers represent insertions. Sequences without indels and with single-base substitution mutation indels do not keep statistics for this chart. d Indel profiles of Anapc2 maternal allele edited embryos by Past-CRISPR. Indel profiles of Anapc2 maternal alleles (left) and paternal alleles (right) are plotted separately. Sequences without indels and with single-base substitution mutation indels do not keep statistics for this chart.

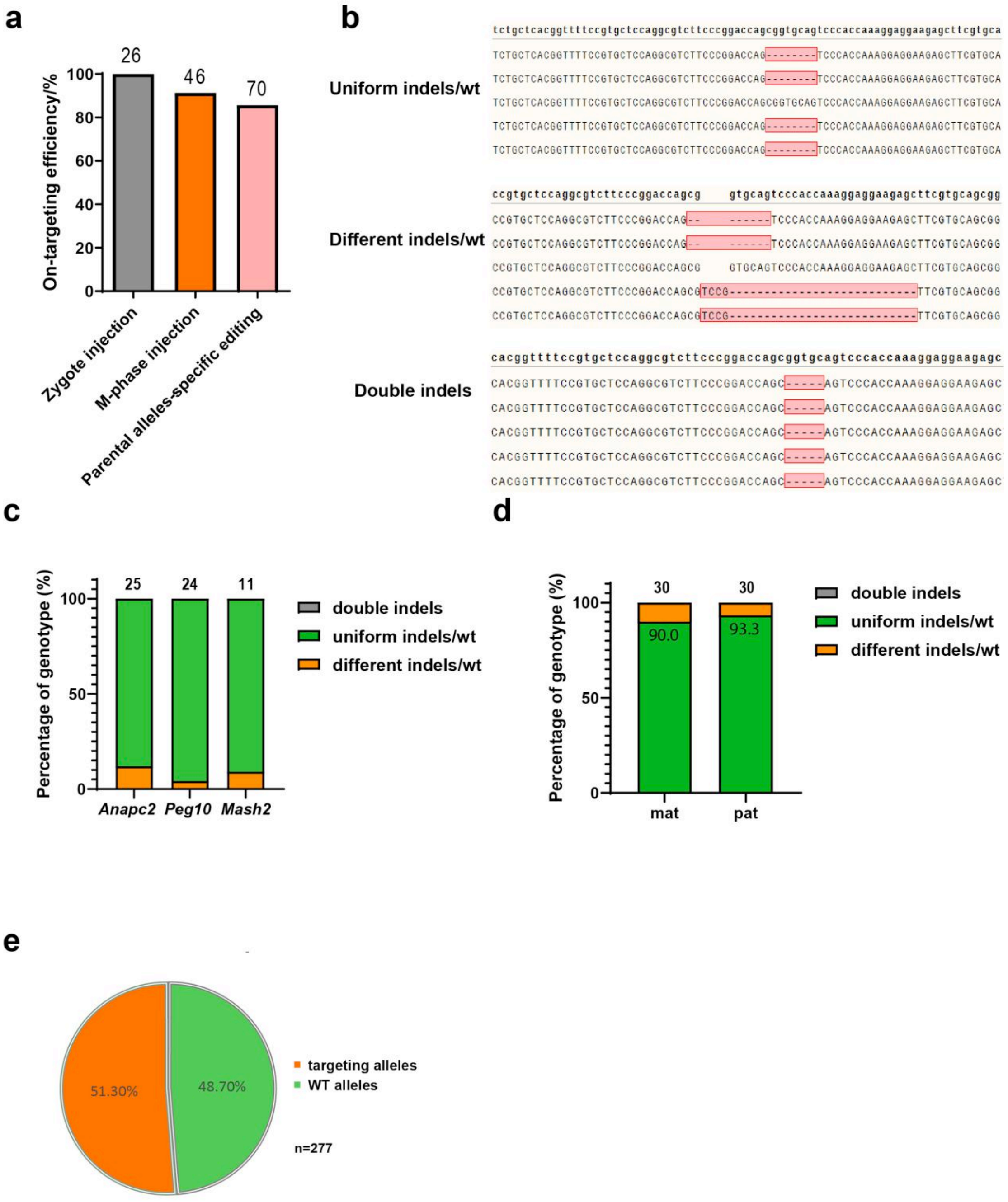

Supplementary figure 4. Comprehensive analysis on the targeting efficiency of Past-CRISPR. **a** Targeting efficiency was compared between the zygote injection embryos, parental allele-specific edited embryos and Cas9 RNP injection in M-phase embryos. **b** Three predicted genotypes in these targeting outcomes of the parental allele-specific edited embryos. Uniform indels/wt represents genotypes contain identical mutation sites and wildtype sites. Different indels/wt represents genotypes contain different mutation sites and wildtype sites. Double indels represents all alleles are mutation sites. **c** Bar charts showing the percentage of the three predicted genotypes in the *Anapc2*, *Peg10* and *Mash2* parental allele-specific edited embryos. Number, total embryos counted.

d Bar charts showing the percentage of the three predicted genotypes in the maternal allele-specific gene edited embryos (Mat) and paternal allele-specific gene knockout embryos (Pat). Number, total embryos counted. **e** Pie charts demonstrating proportions of targeting alleles and wildtype alleles by enlarging the sample size of the parental allele-specific edited embryos (n=70) and for different genes (*Anapc2*, *Peg10*, *Mash2*). Number, total alleles counted.

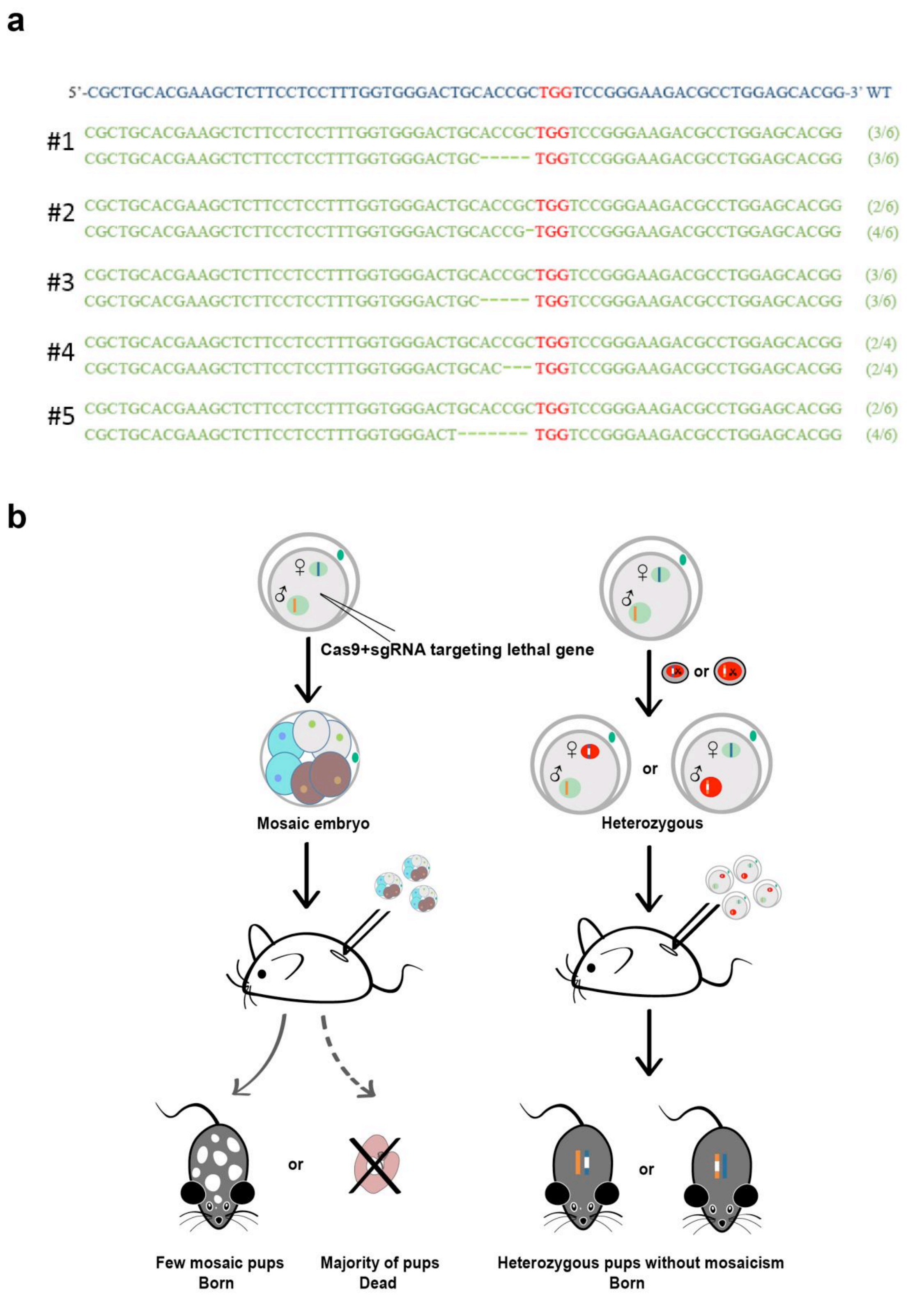

Supplementary figure 5. Generation of lethal gene targeted heterozygous mutant mice rapidly and precisely. a TA cloning sequences of the *Anapc2* target sites from the finger of the heterozygous *Anapc2* mutant mice generated by Past-CRISPR. Sequences of 4-6 alleles reveals the genotype of uniform indels/wt. **b** Schematic. The left panel represents pups generated by zygote injection

methods. Targeting lethal gene, the majority of offspring could not develop to term and few offspring alive shows mosaicism. The right panel represents pups generated by parental allele-specific edited methods, offspring generated is heterozygous mutant without mosaicism.

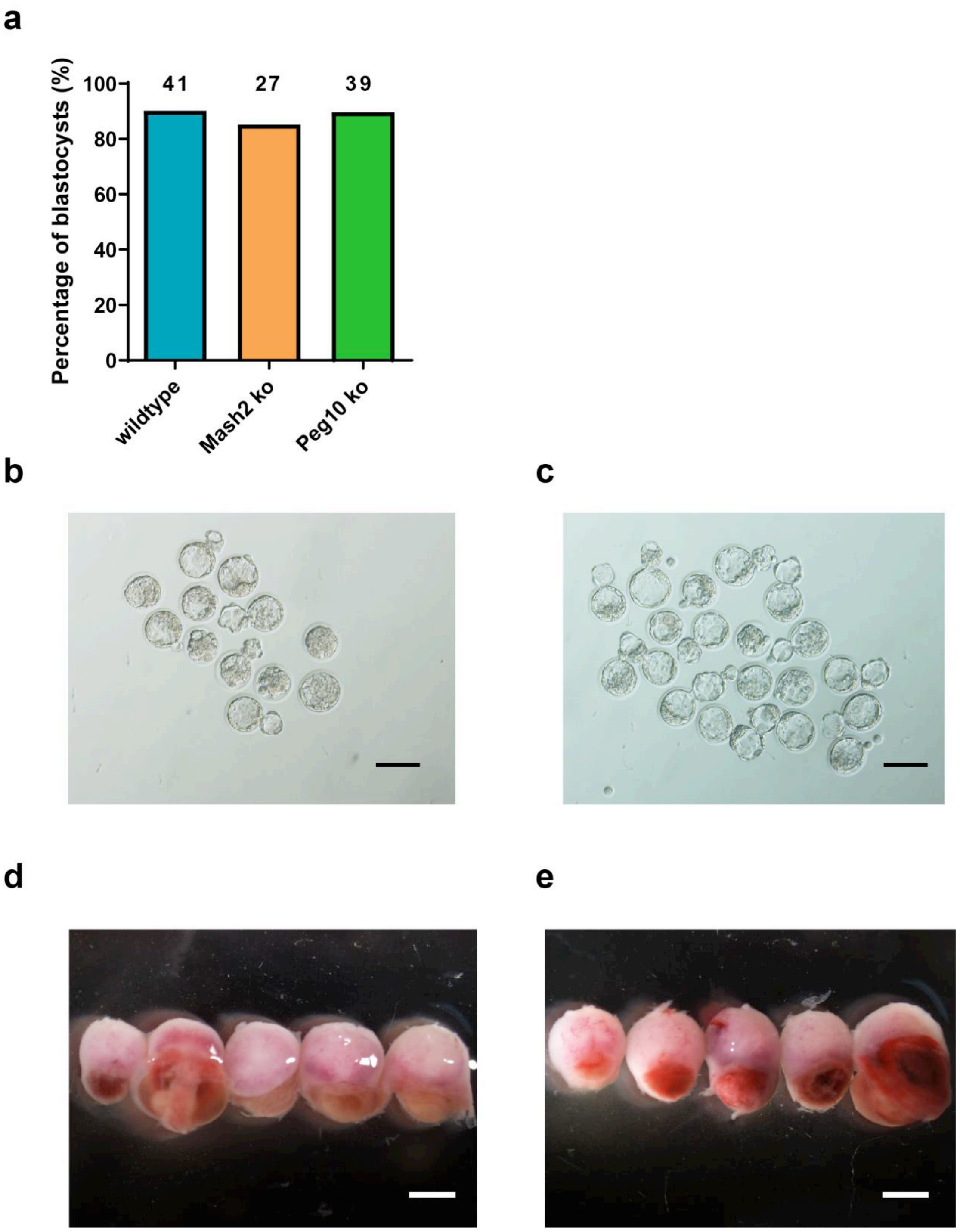

Supplementary figure 6. Development and phenotype of *Peg10* and *Mash2* knockout embryos. **a** Bar charts showing the developmental potential (counted 96h after fertilization) of *Mash2* knockout embryos, *Peg10* knockout embryos and wildtype embryos. Two individual experiments were performed. Source data are provided as a Source Data file. **b,c** Bright fields of *Mash2* knockout and *Peg10* knockout embryos at 3.5 d.p.c., one representative result of three independent experiments is shown. Scale bars: 100 um. **d,e** Representative images showing the remaining

decidual tissue at 11.0 d.p.c. after depleting *Mash2* gene or *Peg10* gene. Embryos have degenerated after depleting *Mash2* or *Peg10* at 11.0 d.p.c.. One representative result of three independent experiments is shown. Scale bars: 1mm.

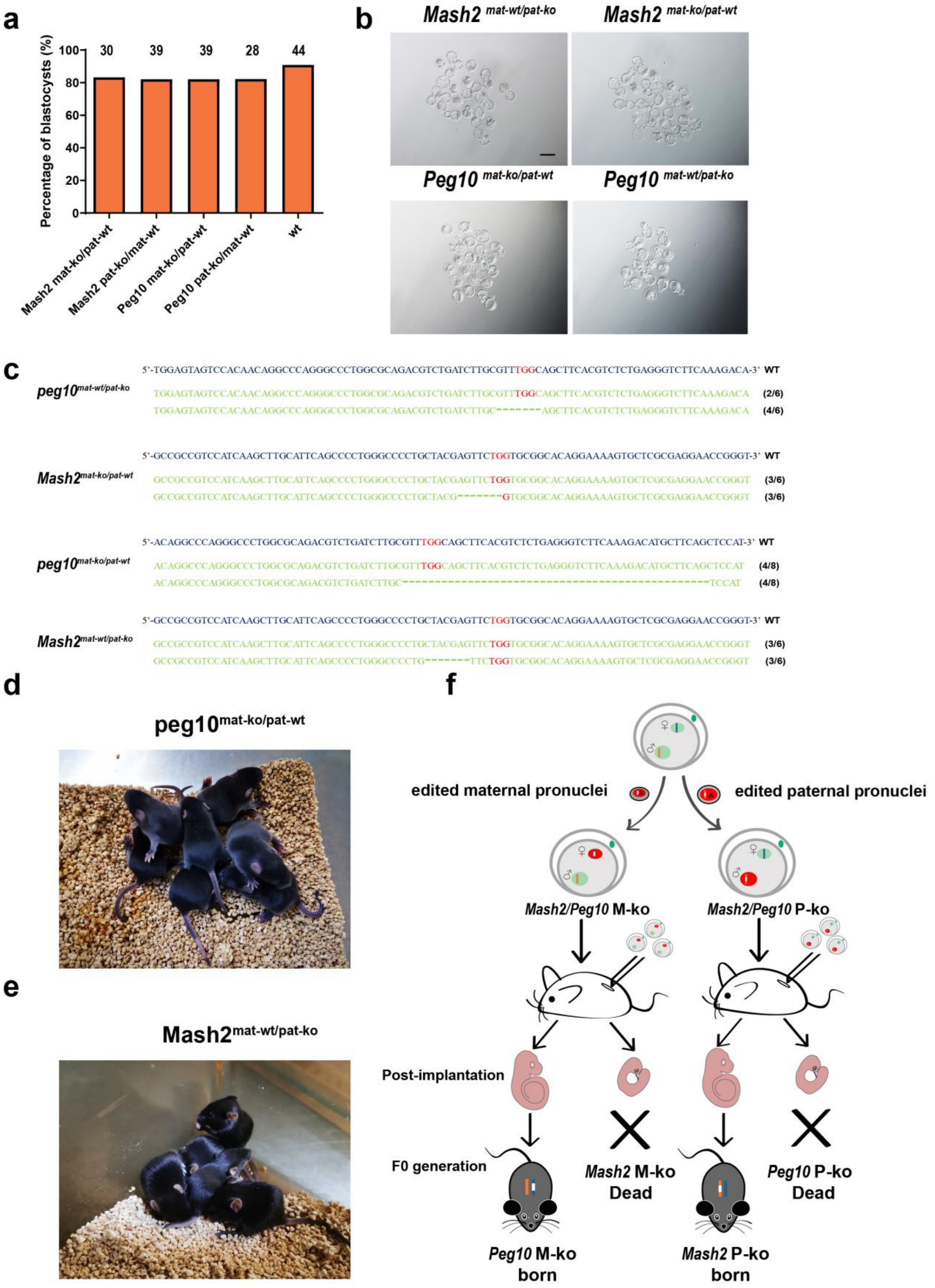

Supplementary figure 7. High-efficient functional verification of *Peg10* and *Mash2*. a Bar charts showing the developmental potential (counted 96h after fertilization) of *Mash2* and *Peg10* parental allele specific knockout embryos and wildtype embryos. Two independent experiments were performed for every types of embryos. Source data are provided as a Source Data file. **b** Bright

fields of *Mash2* and *Peg10* parental allele specific knockout embryos at 4.0 d.p.c., one representative result of three independent experiments is shown. Scale bars: 100um. **c** TA cloning sequencing of total four types of *Peg10* and *Mash2* parental allele specific knockout embryos at 10.0 d.p.c.. Sequences of 6-8 alleles reveals the genotype of uniform indels/wt. **d,e** Representative images show seven *Peg10*$^{mat\text{-}ko/pat\text{-}wt}$ pups generated by *Peg10* maternal allele editing (d) and five *Mash2*$^{mat\text{-}wt/\ pat\text{-}ko}$ pups generated by *Mash2* paternal allele editing (e). **f** Schematic shows the generating process and phenotype outcomes of *Mash2*/*Peg10*$^{mat\text{-}wt/\ pat\text{-}ko}$ and *Mash2*/*Peg10*$^{mat\text{-}ko/pat\text{-}wt}$ embryos and mice by Past-CRISPR methods.

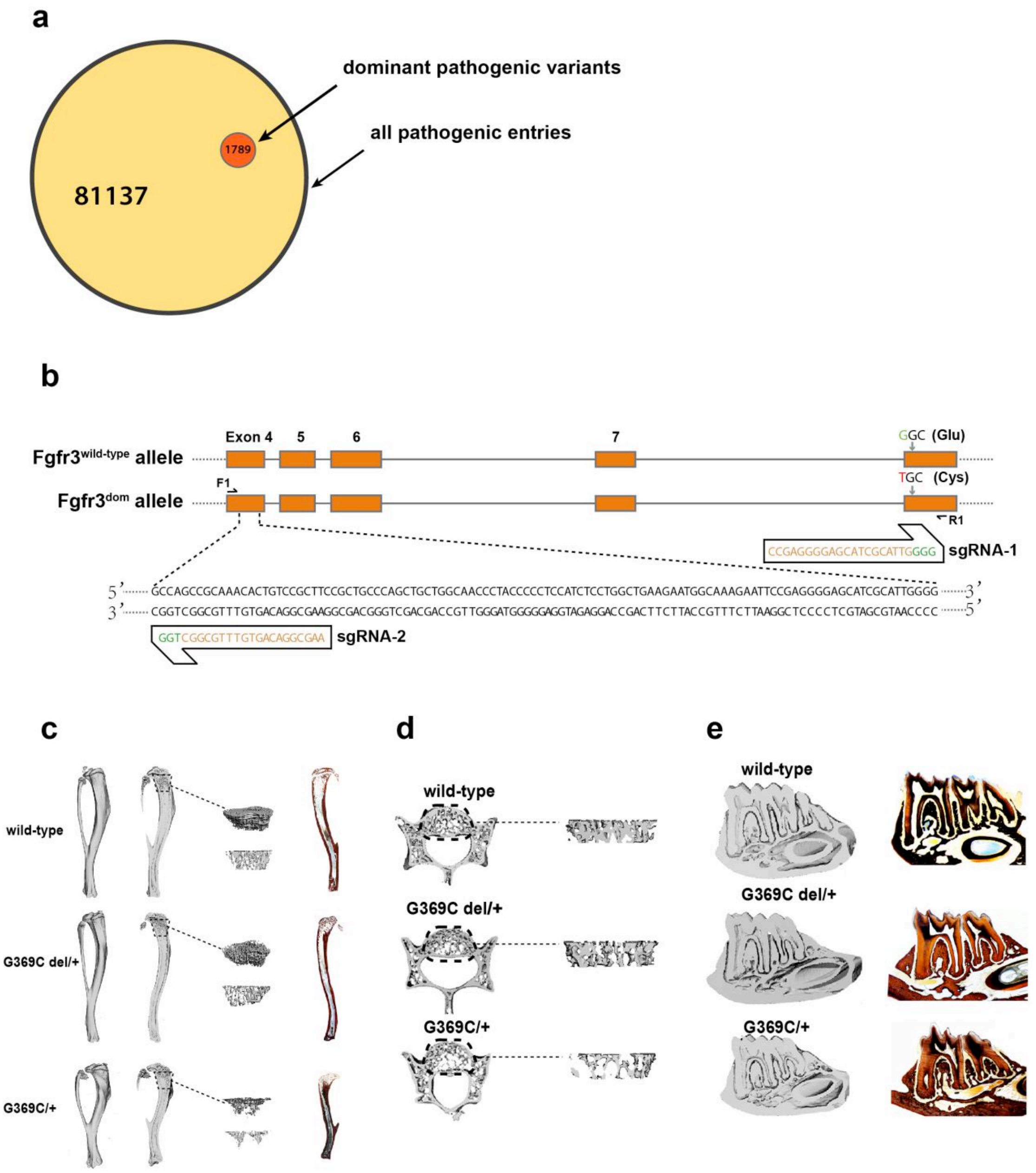

Supplementary figure 8. Identification of bone phenotype and trabecular bone analysis of three types of mice. a All the 70681 known pathogenic pathogenic human genetic variants in the ClinVar database (accessed October, 2019). **b** Schematic diagram of sgRNA design for disrupting the dominant allele of *Fgfr3*. The mutation site is directed by arrows. PAM sites are marked by green nucleotides. F1 and R1 are used for genotyping. **c,d** Trabecular bone quantification analyses and undecalcified bone sections analysis on tibias and vertebras of wildtype (WT), *Fgfr3*$^{\mathrm{G369C\ del/+}}$ and *Fgfr3*$^{\mathrm{G369C/+}}$ mice. Tibias of *Fgfr3*$^{\mathrm{G369C/+}}$ mice exhibit cortical bone thickness, notice sparse bone trabecular and shortened backbone. Vertebras of which exhibit notice sparse bone trabecular and bone morphologic changes. Tibias and Vertebras of *Fgfr3*$^{\mathrm{G369C\ del/+}}$ mice return to nearly normal

level. **e** Trabecular bone quantification analyses and undecalcified bone sections analysis on jaws of wildtype (WT), *Fgfr3*$^{G369C\ del/+}$ and *Fgfr3*$^{G369C/+}$ mice. Tibias of *Fgfr3*$^{G369C/+}$ mice are smaller than wildtype and *Fgfr3*$^{G369C\ del/+}$ mice in size and inhibit shortened incisors and shrunken molars. *Fgfr3*$^{G369C\ del/+}$ mice exhibit normal bone formation.

Supplementary Table 1. Number of distinct mutant alleles in Anapc2 targeted embryos

Cas9/sgRNA	Treatment	Total embryos	Number of mutations								No. mutant embryos carrying indels without frameshift mutations
			0	1	2	3	4	5	6	7	
Cas9 protein+sgRNA	M-phase injection	46	4	3	18	8	6	4	3	0	31
Cas9 mRNA+sgRNA	Zygote injection	17	0	0	4	5	4	3	0	1	10

Supplementary Table 2. Sequencing outcomes of single blastomeres from six one-cell injection embryos

Genotypes of one-cell injection embryos			
Embryo ID	Blastomere ID	Indels	Edit alleles
#1	1.1	5bp ins 8bp del	TCCTTTGGTGGGACT--------TCCAGGGTCCGG
	1.2	14bp del	TCCTTT--------------GCTGGTCCGG
	1.3	13bp del	TCCTTTGGTGGGA-------------CCGGG
	1.4	13bp del	TCCTTTGGTGGGA-------------CCGGG
#2	2.1	13bp del	CCTTTGGTGGGA-------------C
	2.2	14bp del	CCTTTGG--------------TGGTC
#3	3.1	26bp del 1bp mut	CTGCTGCACGAAG--------------------------CGCTGGTCCGG
	3.2	26bp del	CCGCTGCACGAAG--------------------------CGCTGGTCCGG
	3.3	13bp del	TTCCTCCTTTGGTGGGA-------------CCGG
	3.4	3bp del	GGTGGGACTGCA---CTGGTCCGG
#4	4.1	1bp ins 5bp del&3bp del	CTTTGGTGGGACTGA-----CTGGTCC
			CTTTGGTGGGACTGCAC---TGGTCC
	4.2	13bp del	CTTTGGTGGGAC-------------C
	4.3	3bp del&14bp del	CTTTGGTGGGACTGC---GCTGGTCC
			CTTTG--------------CTGGTCC
	4.4	5bp del	CTTTGGTGGGACTGC-----TGGTCC
#5	5.1	3bp del	CTTTGGTGGGACTGCA---CTGGTCC
	5.2	3bp del	CTTTGGTGGGACTGCA---CTGGTCC
	5.3	1bp ins 10bp del	CTTTGGTGGGACTGCA----------AGGG
#6	6.1	13bp del 1bp mut	CATTTGGTGGGA
	6.2	13bp del	CCTTTGGTGGGA-------------CCG
	6.3	13bp del	CCTTTGGTGGGA-------------CCG

Supplementary Table 3. Genotype of PWK/C57 embryos edited by Past-CRISPR

Embryo ID	Parental allele edited	Indels	Edit alleles	Matching SNP	
				T(PWK)	G(C57)
#1	Mat	1bp ins	CTTTGGTGGGACTGCACTCGCTGGT		√
		WT	CTTTGGTGGGACTGCACCGCTGGT	√	
#2	Mat	6bp del	CTTTGGTGGGAC------GCTGGTCCGGGA		√
		WT	CTTTGGTGGGACTGCACCGCTGGT	√	
#3	Mat	4bp ins<br>270bp del	CCAGACGAGTCCCATG--···--TGGTCCG		√
		WT	CTTTGGTGGGACTGCACCGCTGGT	√	
#4	Mat	6bp del	CTTTGGTGGGAC------GCTGGTCCGGGA		√
		WT	CTTTGGTGGGACTGCACCGCTGGT	√	
#5	Mat	1bp ins	CTTTGGTGGGACTGCACACGCTGGTCC		√
		WT	CTTTGGTGGGACTGCACCGCTGGT	√	
#6	Mat	49bp ins	CTTTGGTGGGACTGCACCCACCACGAAGCTCTTCCTCCT TTGGTGGGACTGCTCCTTTGGTGGGACTGCTGGTCCGG		√
		WT	CTTTGGTGGGACTGCACCGCTGGT	√	
#7	Mat	1bp ins	CTTTGGTGGGACTGCACCCGCTGGTCCGGGAA		√
		5bp del	CTTTGGTGGGACTGC-----TGGTCCGGGAAG	√	
		WT	CTTTGGTGGGACTGCACCGCTGGT	√	
#8	Pat	21bp ins	CTTTGGTGGGACTGCACCCACCAAAGGAGGGTGCATCC CGCTGGTCC	√	
		WT	CTTTGGTGGGACTGCACCGCTGGT		√
#9	Pat	2bp ins	CTTTGGTGGGACTGCACCGCGCTGGTC	√	
		WT	CTTTGGTGGGACTGCACCGCTGGT		√
#10	Pat	1bp del	CTTTGGTGGGACTGCAC-GCTGGTCCGGG	√	
		WT	CTTTGGTGGGACTGCACCGCTGGT		√
#11	Pat	13bp ins<br>1bp del	CTTTGGTGGGACTGCTCCAGGTGTCTTC-CCGCTGG	√	
		WT	CTTTGGTGGGACTGCACCGCTGGT		√
#12	Pat	16bp del	CTTTGGT----------------CCGGGAAGAC	√	
		5bp del	CTTTGGTGGGACTGC-----TGGTCCGGGAAGAC		√
		WT	CTTTGGTGGGACTGCACCGCTGGT		√
#13	Pat	8bp del	CTTTGGTGGGACT--------GGTCCGGGAAGACG	√	
		WT	CTTTGGTGGGACTGCACCGCTGGT		√
#14	Pat	1bp ins	CTTTGGTGGGACTGCACCCGCTGGTCC	√	
		WT	CTTTGGTGGGACTGCACCGCTGGT		√
#15	Pat	12bp ins	CTTTGGTGGGACTGCACCAAAGGAGGAAGAGCTGGTCC GGGA	√	
		WT	CTTTGGTGGGACTGCACCGCTGGT		√

Supplementary Table 4. Summary of Past-CRIPSR-mediated targeting.

Gene	Group	Embryos for sequencing	Mutant embryos	NHEJ efficiency (%)	Monoallelic targeting embryos	Monoallelic targeting efficiency (%)	Genetic mosaicism efficiency (%)	Embryos transferred (recipients) (n)	Embryos(arrested embryos) at 10.0 d.p.c.(n)	New borns(n)	Heterozygous mice without mosaicism (n)	Surviving adult mutant mice(n)
Anapc2	Mat-edited	13	13	100	11	84.61	15.38	60(3)	——	8	5	5
	Pat-edited	12	12	100	11	91.66	8.33	——	——	——	——	——
Anapc2	traditional zygote injection	17	17	100	0	0	76.5	——	——	——	——	——
Anapc2	MII injection	46	42	91.3	1	2.38	45.7	——	——	——	——	——
Peg10	Mat-edited	13	11	84.62	11	100	0	100(6)	6(0)	9	7	7
	Pat-edited	17	13	76.47	12	92.31	5.88	80(5)	13(13)	——	——	——
Peg10	traditional zygote injection	9	9	100	0	0	100	——	——	——	——	——
Mash2	Mat-edited	9	6	66.67	5	83.33	11.11	50(3)	6(6)	——	——	——
	Pat-edited	6	5	83.33	5	100	0	100(7)	7(0)	7	5	5

Supplementary Table 5. SgRNA target sites and primer used for target gene PCRs

Anapc2-1	CTTTGGTGGGACTGCACCGC**TGG**
Anapc2-2	GCAATATTGCCACGGAGTTC**TGG**
Mash2-1	TGGGCCCCTGCTACGAGTTC**TGG**
Mash2-2	CGAGCGCAACCGCGTAAAGC**TGG**
Peg10-1	CAGACGTCTGATCTTGCGTT**TGG**
Peg10-2	GATCGGCCGTGCCGCCCGCT**GGG**
Tyr	GTTATGGCCGATAGGTGCAT**TGG**
Fgfr3-sgRNA1	CCGAGGGGAGCATCGCATTG**GGG**
Fgfr3-sgRNA2	AAGCGGACAGTGTTTGCGGC**TGG**
Anapc2-F	CAGATTTCCCGCGACTTTGC
Anapc2-R	AGAGAAACCGCAGCATGGTTA
Mash2-F	CACGAGAGTACCACGCTTCC
Mash2-R	GCGTCTCCACCTTACTCAGC
Peg10-F	TGATGAACGCAGACACGA
Peg10-R	AGGCTCTGGGTGAAGGAT
Tyr-outer-F	GTTATCCTCACACTACTTCTG
Tyr-outer-R	GTAATCCTACCAAGAGTCTCA
Tyr-inner-F	TCCTCACACTACTTCTGATG
Tyr-inner-R	GTCTCAAGATGGAAGATCAC
Fgfr3-KO-F	AATCCAGAAGGGTAAACAGG
Fgfr3-KO-R	AAGGTACTGGTTCGAGGT
Fgfr3-pointmut-F	CTCCAAGTATCCCAGGTCC
Fgfr3-pointmut-R	AGGAGACACGAGGCAGGA
Fgfr3-all-F1	GGAGCGAATGGATAAGAAA
Fgfr3-all-R1	TGGAAACAGAGGCAGTGAG
Fgfr3-all-F2	TAGGCTCTATCCCCGGCTG
Fgfr3-all-R2	GCAAGGTGTGGAGCCAAG
Cas9-F	TAATACGACTCACTATAGGGAGAATGGACTATAAGGACCACGAC
Cas9-R	GCGAGCTCTAGGAATTCTTAC
