# Supplementary material for: Precise allele-specific genome editing by spatiotemporal control of CRISPR-Cas9 via pronuclear transplantation
Source: Nat Commun. 2020 Sep 14;11:4593. doi: 10.1038/s41467-020-18391-y (PMC7490392; doi:10.1038/s41467-020-18391-y)
Supplement: Supplementary file 2 — Reporting Summary [file 41467_2020_18391_MOESM2_ESM.pdf]

nature research

Corresponding author(s): Shao rong Gao

Last updated by author(s): Aug 10, 2020

Reporting Summary

Nature Research wishes to improve the reproducibility of the work that we publish. This form provides structure for consistency and transparency in reporting. For further information on Nature Research policies, see Authors & Referees and the Editorial Policy Checklist .

Statistics

For all statistical analyses, confirm that the following items are present in the figure legend, table legend, main text, or Methods section.

n/a	Confirmed	
☐	☒	The exact sample size (*n*) for each experimental group/condition, given as a discrete number and unit of measurement
☐	☒	A statement on whether measurements were taken from distinct samples or whether the same sample was measured repeatedly
☐	☒	The statistical test(s) used AND whether they are one- or two-sided<br>*Only common tests should be described solely by name; describe more complex techniques in the Methods section.*
☒	☐	A description of all covariates tested
☒	☐	A description of any assumptions or corrections, such as tests of normality and adjustment for multiple comparisons
☐	☒	A full description of the statistical parameters including central tendency (e.g. means) or other basic estimates (e.g. regression coefficient) AND variation (e.g. standard deviation) or associated estimates of uncertainty (e.g. confidence intervals)
☐	☒	For null hypothesis testing, the test statistic (e.g. *F*, *t*, *r*) with confidence intervals, effect sizes, degrees of freedom and *P* value noted<br>*Give P values as exact values whenever suitable.*
☒	☐	For Bayesian analysis, information on the choice of priors and Markov chain Monte Carlo settings
☒	☐	For hierarchical and complex designs, identification of the appropriate level for tests and full reporting of outcomes
☒	☐	Estimates of effect sizes (e.g. Cohen's *d*, Pearson's *r*), indicating how they were calculated

Our web collection on statistics for biologists contains articles on many of the points above.

Software and code

Policy information about availability of computer code

Data collection	No software was used in this analysis.
Data analysis	We used NCBI and CRISPOR for designing the primer and sgRNA, and SnapGene (v2.3.2) for analyzing Sanger sequencing data. Quantitative analysis were performed by GraphPad Prism 8.0. TIDE analysis were performed by http://shinyapps.datacurators.nl/tide/

For manuscripts utilizing custom algorithms or software that are central to the research but not yet described in published literature, software must be made available to editors/reviewers. We strongly encourage code deposition in a community repository (e.g. GitHub). See the Nature Research guidelines for submitting code & software for further information.

Data

Policy information about availability of data

All manuscripts must include a data availability statement. This statement should provide the following information, where applicable:
- Accession codes, unique identifiers, or web links for publicly available datasets
- A list of figures that have associated raw data
- A description of any restrictions on data availability

All relevant data are reported in the main text or Supplementary Information. The source data underlying Figs 3e, 4b and 4h and Supplementary Figs 1b, 6a, 7a are provided as Source Data files. Any additional data relevant to this manuscript are available from the authors upon reasonable request.

Field-specific reporting

Please select the one below that is the best fit for your research. If you are not sure, read the appropriate sections before making your selection.

- [x] Life sciences
- [ ] Behavioural & social sciences
- [ ] Ecological, evolutionary & environmental sciences

For a reference copy of the document with all sections, see nature.com/documents/nr-reporting-summary-flat.pdf

Life sciences study design

All studies must disclose on these points even when the disclosure is negative.

Sample size	Sample sizes were determined without statistical measures, but based on prior experience with the specific experiments and widely used sizes in relevant publications within this field of research in order to ensure that it will be appropriate for statistical analysis. See Methods for detail.
Data exclusions	No data was exluded from the analyses
Replication	All data could be reproduced, and most experiments and analyses presented were the result of at least two independent biological replicates. All attempts at replication were successful.
Randomization	This study did not include complex treatment conditions, and all embryos and pups mentioned on results were manipulated and analyzed under identical conditions.
Blinding	Experiments execution, data collection and result analysis were usually carried out by the same person, therefore no blinding was used.

Reporting for specific materials, systems and methods

We require information from authors about some types of materials, experimental systems and methods used in many studies. Here, indicate whether each material, system or method listed is relevant to your study. If you are not sure if a list item applies to your research, read the appropriate section before selecting a response.

Materials & experimental systems

n/a	Involved in the study	
☐	☒	Antibodies
☒	☐	Eukaryotic cell lines
☒	☐	Palaeontology
☐	☒	Animals and other organisms
☒	☐	Human research participants
☒	☐	Clinical data

Methods

n/a	Involved in the study	
☒	☐	ChIP-seq
☒	☐	Flow cytometry
☒	☐	MRI-based neuroimaging

Antibodies

Antibodies used	Mouse anti-Cas9 1:10 (AB_2793760, Active motif, Catalog No: 61758)
Validation	Host: Mouse<br>Antibody type: Monoclonal<br>Citation:<br>Stuart, W. D., Guo, M., et al. (2020), 'CRISPRi-mediated functional analysis of lung disease-associated loci at non-coding regions.', NAR Genom Bioinform, 2 (2), pp. lqaa036<br>Fuentes, D. R., Swigut, T., et al. (2018), 'Systematic perturbation of retroviral LTRs reveals widespread long-range effects on human gene regulation.', Elife, 7<br>Liu, X. S., Wu, H., et al. (2018), 'Rescue of Fragile X Syndrome Neurons by DNA Methylation Editing of the FMR1 Gene.', Cell, 172 (5), pp. 979-992.e6

Animals and other organisms

Policy information about studies involving animals; ARRIVE guidelines recommended for reporting animal research

Laboratory animals	Laboratory mice (Mus musculus) were used in this study. The specific patrogen-free mice were housed in the animal facility of Tongji University, Shanghai, China. The use and care of animals complied with the guideline of the Tongji University Guide for the Use of Laboratory Animals. The following backgrounds were used: eight-week-old C57BL/6, DBA/2, ICR, PWK and BDF1. The

following specific strains were used: Fgfr3 G369C were acquired from Prof. Lin Chen. Adult female mice (8 weeks old) were used for breeding and superovulation. Adult male mice (8 weeks old) were used for breeding and mating with females or collecting sperm. 2-cell or embryos or blastocysts were transferred into ICR female mice (8-10 week). Genotyping was performed from birth to P20.

Wild animals	No wild animals were used in this study.
Field-collected samples	No field collected samples were used in this study.
Ethics oversight	The use and care of animals complied with the guideline of the Biological Research Ethics Committee of Tongji University.

Note that full information on the approval of the study protocol must also be provided in the manuscript.
