# Supplementary material for: Precise allele-specific genome editing by spatiotemporal control of CRISPR-Cas9 via pronuclear transplantation
Source: Nat Commun. 2020 Sep 14;11:4593. doi: 10.1038/s41467-020-18391-y (PMC7490392; doi:10.1038/s41467-020-18391-y)
Supplement: Supplementary file 4 — Description of Additional Supplementary Files [file 41467_2020_18391_MOESM4_ESM.pdf]

Title: Supplementary Data 1

Description: Clinvar Database, pathogenic entries.

Title: Supplementary data 2

Description: Clinvar Database, dominant pathogenic variants screening.
